# Immobilization of *Lathyrus cicera* Amine Oxidase on Magnetic Microparticles for Biocatalytic Applications

**DOI:** 10.3390/ijms23126529

**Published:** 2022-06-10

**Authors:** Elisa Di Fabio, Antonia Iazzetti, Alessio Incocciati, Valentina Caseli, Giancarlo Fabrizi, Alberto Boffi, Alessandra Bonamore, Alberto Macone

**Affiliations:** 1 Department of Biochemical Sciences “Alessandro Rossi Fanelli”, Sapienza University of Rome, Piazzale Aldo Moro 5, 00185 Rome, Italy; elisa.difabio@uniroma1.it (E.D.F.); alessio.incocciati@uniroma1.it (A.I.); alberto.boffi@uniroma1.it (A.B.); 2 Department of Chemistry and Technology of Drugs, Sapienza University of Rome, Piazzale Aldo Moro 5, 00185 Rome, Italy; antonia.iazzetti@unicatt.it (A.I.); giancarlo.fabrizi@uniroma1.it (G.F.); 3 Dipartimento di Scienze Biotecnologiche di Base, Cliniche Intensivologiche e Perioperatorie, Università Cattolica del Sacro Cuore, L.go Francesco Vito 1, 00168 Rome, Italy; 4 Center for Life Nano Science@Sapienza, Istituto Italiano Di Tecnologia, V.le Regina Elena 291, 00161 Rome, Italy; valentina.caseli@iit.it

**Keywords:** enzyme immobilization, amine oxidase, magnetic particles, aldehydes, biocatalysis, oxidative deamination, primary amines

## Abstract

Amine oxidases are enzymes belonging to the class of oxidoreductases that are widespread, from bacteria to humans. The amine oxidase from *Lathyrus cicera* has recently appeared in the landscape of biocatalysis, showing good potential in the green synthesis of aldehydes. This enzyme catalyzes the oxidative deamination of a wide range of primary amines into the corresponding aldehydes but its use as a biocatalyst is challenging due to the possible inactivation that might occur at high product concentrations. Here, we show that the enzyme’s performance can be greatly improved by immobilization on solid supports. The best results are achieved using amino-functionalized magnetic microparticles: the immobilized enzyme retains its activity, greatly improves its thermostability (4 h at 75 °C), and can be recycled up to 8 times with a set of aromatic ethylamines. After the last reaction cycle, the overall conversion is about 90% for all tested substrates, with an aldehyde production ranging between 100 and 270 mg depending on the substrate used. As a proof concept, one of the aldehydes thus produced was successfully used for the biomimetic synthesis of a non-natural benzylisoquinoline alkaloid.

## 1. Introduction

The last few decades have witnessed a dramatic development of biocatalysis as a competitive and cost-effective technology for the synthesis of fine chemicals and active pharmaceutical intermediates [1,2,3,4,5,6]. The use of enzymes from both natural sources or produced in recombinant form, as raw or highly purified extracts, is also becoming increasingly common [7,8,9]. Enzymes can be used in cascades that mimic synthetic biochemical pathways, or they can replace some steps in the total synthesis of natural and non-natural molecules with high structural complexity [10,11,12,13,14]. However, the use of enzymes can be challenging on a large scale due to their high production costs, low operational stability, and difficulties in recovering them from the reaction mixtures [15]. Thus, a promising way to improve their performance is the immobilization onto solid supports, which also has the advantage of allowing their recovery and recycling [16,17,18]. Depending on the support on which they are immobilized, enzymes often improve their biochemical and kinetic properties, and, being recyclable, they can also be used in the scale-up of synthetic processes [19,20]. For quite a long time, successful immobilization of biocatalysts has been largely confined to hydrolytic enzymes [21,22,23], but this scenario is changing with the development of enzymes for a wider range of biotransformations, including asymmetric reduction, carbon–carbon bond formation, and oxidation [24,25,26]. In this regard, increasing attention is paid to amine oxidases, enzymes belonging to the class of oxidoreductases that are widespread in nature (from bacteria to humans). These enzymes have recently appeared in the landscape of biocatalysis: they have shown good potential in both amine resolution and functionalization [27] and in the green synthesis of aldehydes [28]. To date, however, very few of them (namely those using FAD as a cofactor) have been immobilized on solid supports, mainly to develop biosensors for diagnostic purposes [29,30]. In this paper, we focus our attention on a plant Cu-containing amine oxidase (LCAO, *Lathyrus cicera* amine oxidase), which our research group has been studying for the biocatalytic productions of aldehydes. In nature, LCAO catalyzes the oxidative deamination of primary biogenic amines (i.e., putrescine and cadaverine) into the corresponding aldehydes [31]. We have shown that this enzyme has a relaxed substrate specificity, as it can convert a wide range of aliphatic and aromatic amines [28]. Although very promising, this system has some limitations, which include the low solubility of the oxygen co-substrate of the reaction, and the possible inactivation of the enzyme that may occur at high aldehyde concentration. One of the ways to overcome these issues could be the immobilization of the enzyme. Typically, immobilization improves the overall catalytic performance, allowing enzyme recycling, and a reduction in the overall process costs [32]. Among the different support materials, magnetic nanoparticles are considered the future of enzyme immobilization, due to their exceptional ease of handling, recovery, and reuse [33,34]. Thus, the present work aims at evaluating the activity of LCAO immobilized on various supports, including magnetic particles. As a proof of concept, we showed that the aldehydes produced with the immobilized enzyme can be easily used for the biomimetic synthesis of new benzylisoquinolines, structurally complex alkaloids that typically require a challenging chemical synthetic sequence [35].

## 2. Results and Discussion

### 2.1. Immobilization of LCAO

Cu-containing amine oxidases are enzymes highly expressed in *Leguminosae* sp. Among them, *Lathyrus cicera* is an extremely abundant source of this enzyme, where it is produced at high yields and accumulates in the periplasmic space of the etiolated sprouts. In a recent paper, we demonstrated that LCAO can be purified using an easily scalable chromatography-free protocol [28]. The purified enzyme is naturally active on biogenic primary amines, but it can be also used for the biocatalytic synthesis of a wide range of aliphatic and aromatic aldehydes, starting from the corresponding amines. Typically, biotransformation of non-natural substrates requires more enzyme with respect to the natural substrates, and this could be a problem when considering a large-scale synthesis. In this regard, LCAO immobilization may overcome this issue, by allowing its recycling/reuse, and at the same time, by avoiding the possible inactivation due to the accumulation of the aldehyde. Optimal immobilization strategies have to be tailored for each specific enzyme; thus, we tested which solid support was the most suitable for immobilizing LCAO. As a first step, we selected a series of resins and differently functionalized magnetic and non-magnetic particles, to test the binding capacity of the enzyme and its activity. Specifically, we chose different types of solid supports: a DEAE Sepharose resin, chloromethyl latex microparticles, and three different magnetic beads (COOH- or NH_2_-functionalized microparticles (COOH-MMPs and NH_2_-MMPs), and TurboBeads amine nanoparticles).

**Figure 1:**
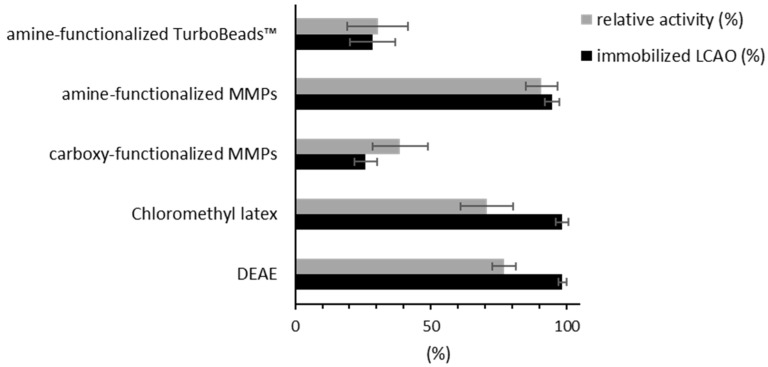
LCAO immobilization on different supports. Data are presented as mean ± SD (*n* = 3).

The surface lysine residues of the enzyme are used to covalently bind the selected supports using different strategies. Glutaraldehyde and 1-ethyl-3-(3-dimethylaminopropyl) carbodiimide hydrochloride (EDC) crosslinkers were used for amine-functionalized supports (DEAE, NH_2_-MMPs, and TurboBeads amine nanoparticles) and COOH-MMPs, respectively; chloromethyl latex nanoparticles do not require crosslinker agents as they display on their surface chloromethyl groups which yield a stable covalent bond with amino groups of LCAO, in a one-step process under mild aqueous conditions. As shown in Figure 1, all the selected supports were able to bind the enzyme which, in all cases, was active.

Greater difficulties were encountered with the TurboBeads magnetic nanoparticles that, due to the low diameter (≤50 nm), are quite difficult to handle and tend to aggregate. This results in a low degree of protein binding, even though the linked enzyme is still active. A similar result was obtained when using the COOH-MMPs. In this case, although the relative activity is higher than expected based on the immobilized enzyme, this system is not convenient due to the large loss of protein that occurs during the crosslinking process. LCAO immobilization occurred efficiently on all the other tested solid supports. On chloromethyl latex nanoparticles and DEAE Sepharose resin very similar results were obtained: about 100% of the enzyme was bound with an enzymatic activity of about 70–80%. Both systems have advantages: chloromethyl latex beads are easy to prepare while DEAE resin can be packed on a column to generate a kind of flow reactor, in which the amino substrate present in the mobile phase can be converted into the aldehyde by the crosslinked enzyme. The best performance in terms of immobilization and activity was achieved with NH_2_-MMPs. In this case, the amount of immobilized enzyme is comparable to chloromethyl latex and DEAE resin, while the relative activity is definitely higher (>90%). These results can be explained by the use of glutaraldehyde as the crosslinker: it typically promotes the formation of multipoint bonds with the enzyme while allowing it to maintain high conformational mobility, likely mimicking its free form. The superparamagnetic property of these micro-sized magnetic beads is useful because individual microparticles become magnetized only when exposed to an external magnetic field, but no magnetization occurs when the field is removed. These unique features can be exploited for enzyme separation from the reaction mixture and its reuse, making them competitive especially for large-scale industrial uses. Given these results, the NH_2_-MMPs were selected as the support of choice for LCAO immobilization.

**Figure 2:**
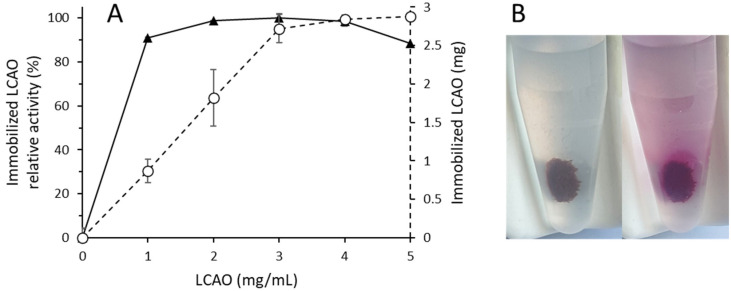
LCAO immobilization on NH_2_-MMPs. (**A**) Effect of LCAO concentration on the immobilization on NH_2_-MMPs (data are presented as the mean ± SD of two experiments). (**B**) LCAO immobilized on NH_2_-MMPs in the absence (left) or the presence (right) of the reagents used for the peroxidase coupled assay. The purple halo around the microparticles grouped by the effect of the magnet indicates that LCAO is immobilized on them.

LCAO-catalyzed oxidative deamination can be easily followed by monitoring hydrogen peroxide by a spectrophotometric assay coupled with peroxidase. The latter, in the presence of AAP and DCHBS, generates a typical purple-colored adduct. This assay can be promptly used to check LCAO immobilization on the surface of MMPs. As shown in Figure 2B, the solution turns purple starting from the particles that gather when a magnetic field is applied.

Alongside these qualitative data, we carried out a quantitative analysis of the immobilization process. The immobilization was performed according to the manufacturer’s instructions, starting from a fixed concentration of particles (10 mg) and glutaraldehyde (10%), varying the amount of enzyme. The parameters used to evaluate the efficiency of the process were the amount of protein bound and its relative activity. Soluble LCAO activity at its optimum (pH 7, 25 °C) was considered 100%. As shown in Figure 2A, we tested enzyme concentrations ranging from 1 to 5 mg/mL measuring at the same time the activity of immobilized LCAO.

In our experimental setting, MMPs are saturated using 3 mg of protein. A further increase in enzyme concentration did not improve the immobilization yield. In addition, in the range of 1–4 mg, the relative activity of the immobilized LCAO was close to 100%. Further increase in enzyme concentration resulted in a 15% loss of LCAO activity that may be explained by reduced accessibility of the substrate to the active site, probably due to a crowding of the enzyme on the surface of the support. Based on these results, the best immobilization yield is achieved by using 3 mg of the free enzyme. The LCAO-MMPs thus obtained were used for the performance study.

### 2.2. Characterization and Performance Study of LCAO-MMPs

**Figure 3:**
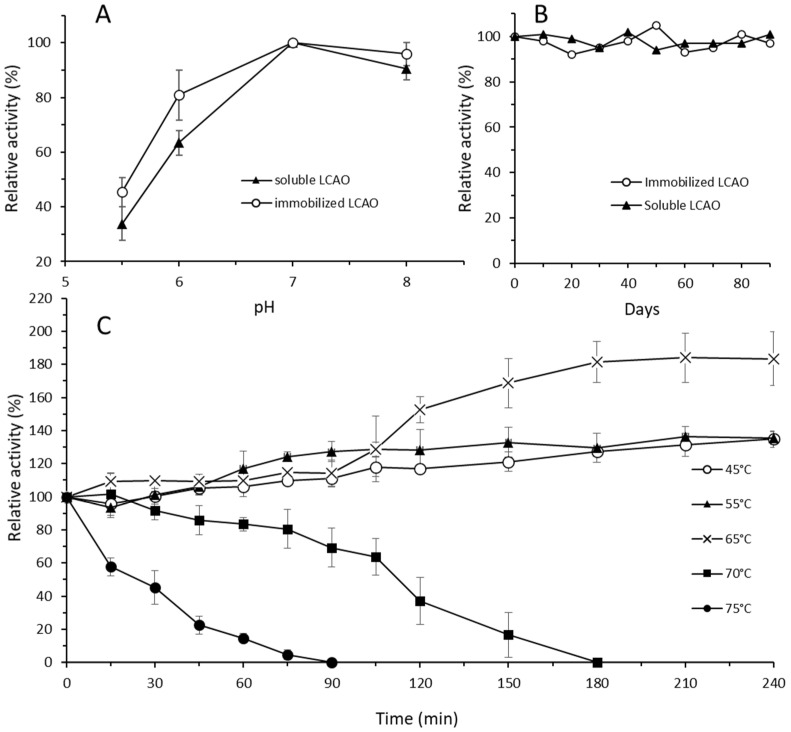
Effect of immobilization of LCAO on reaction pH (**A**), storage stability (**B**), and thermostability (**C**). Data are presented as mean ± SD (*n* = 3).

The effect of reaction pH on the relative activity of both soluble and immobilized enzymes was investigated at different pH values (Figure 3A). As previously reported [28], soluble LCAO has an optimum pH towards its natural substrate putrescine between 7 and 8 while is significantly less active at pH 5.5 and 6. The immobilized enzyme does not show a shift in the optimum pH, while at pH different from 7, the activity is always higher than in the free enzyme (T-test, *p* < 0.05). This might be due to the change in the electrostatic charge of the enzyme after immobilization. Based on these results, the performance study was carried out at this pH value.

The enzyme immobilization method was reliable as three different batches of immobilized enzyme prepared by the procedure described in Section 4 gave comparable results in terms of crosslinked protein (94.57% ± 2.6) and relative activity (90.67% ± 5.86) (Figure 1). As shown in Figure 3B, both soluble and immobilized LCAO stored at 4 °C retain their activity for up to 90 days. These results also prove that there is no protein leakage over time.

In a previous study [28], we have shown that LCAO is stable at 50–60 °C up to 1 h and here we checked whether the immobilized enzyme retains this thermal stability profile. The results are shown in Figure 3C, where the stability is measured in a temperature range of 45–75 °C.

The immobilization procedure resulted in enhanced thermal stability of LCAO, which means an increase in the resistance of the immobilized enzyme towards heat-induced conformational changes. Immobilized LCAO is stable for at least 4 h up to 65 °C, after 2 h at 70 °C it still retains 50% activity, and it is still 50% active after 30 min at 75 °C. This might be explained because the glutaraldehyde treatment enables the formation of covalent bonds that restrict the enzyme unfolding through multipoint linkage. In addition, a 35% enhancement of apparent activity is observed after 4 h at 45 and 55 °C, which further increases to 83% when the enzyme is incubated at 65 °C. This might be the result of specific interactions between magnetic microparticles and enzymes, substrates, or reaction media that could occur at higher temperatures.

**Figure 4:**
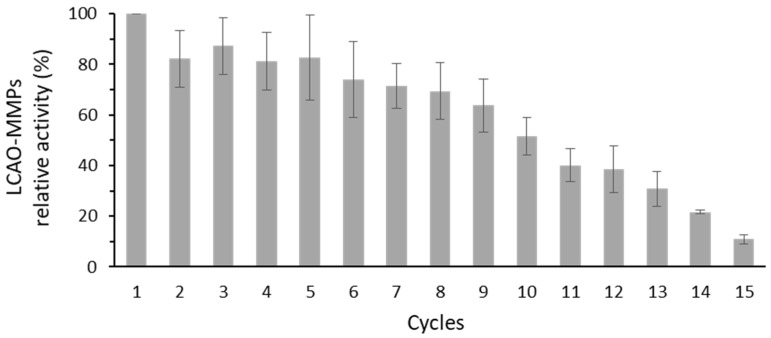
Reusability of LCAO immobilized on NH_2_-MMPs. The reaction was carried out with putrescine as a substrate in the presence of catalase. Data are presented as mean ± SD (*n* = 3).

One of the main reasons for the immobilization of an enzyme for biocatalytic purposes is its reusability and this is important to reduce the overall costs in any industrial application. While free LCAO can be used once, immobilized LCAO can be recycled efficiently. As shown in Figure 4, the enzyme can be reused up to 15 times with its natural substrate putrescine (10.87 ± 1.83% relative activity at cycle 15).

The loss of activity observed during the cycles could be due to the partial inactivation of the enzyme caused by the accumulation of the aldehyde product which can react with the amino groups on the surface of the enzyme itself. However, the immobilized enzyme is more stable with respect to the free one probably because these surface amino groups are already involved in the crosslinking with the MMPs.

### 2.3. Biocatalytic Application of the Immobilized LCAO

**Scheme 1:**
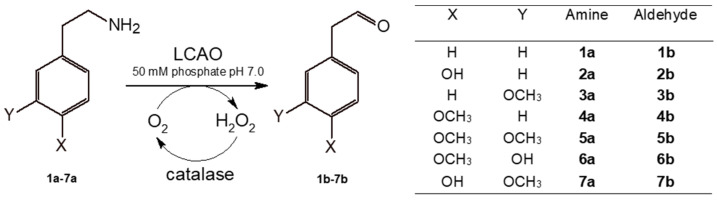
Biocatalytic conversion of primary amines (compounds **1a**–**7a**) into the corresponding aldehydes (compounds **1b**–**7b**) in the presence of LCAO and catalase.

The greater stability of the immobilized enzyme paves the way for its better use for biocatalytic purposes. Free LCAO has a relaxed substrate specificity, being able to process variously substituted aromatic and aliphatic primary amines [28]. In this paper, we aim to test whether the immobilized enzymes can be used for the biocatalytic production of aldehydes starting from a selection of aromatic ethylamines according to Scheme 1.

As a first step, we determined the Michaelis–Menten constant of the immobilized enzyme towards compounds **1a**–**7a** comparing them with the natural substrate putrescine. The Km values are reported in Table 1 and compared with those previously obtained with the free enzyme.

The immobilized LCAO is active on all the substrates being able to convert all the amines tested in the corresponding aldehydes. Km values for LCAO-MMPs are in the same low millimolar range of free enzyme and the small differences observed may be due to a series of factors including stabilization of the enzyme in more active conformations, different accessibility to the catalytic site, and different diffusion rate of substrates on the surface of the nanoparticle.

Based on these data, we tested whether immobilized LCAO could be eligible for scaling up the biocatalytic production of more complex aldehydes (Scheme 1). In a previous paper [28], we showed that LCAO can oxidatively deaminate a variety of aliphatic and aromatic primary amines with almost total conversion. In that case, we used an amine concentration equal to 5 mM as the accumulation of reaction product aldehyde tended to inactivate the enzyme. To overcome this drawback, more units of LCAO were added at regular intervals until the reaction was completed. The immobilized enzyme is less susceptible to inactivation by the accumulation of the reaction products, probably because its surface amino groups are instead used to bind to the magnetic microparticle.

**Figure 5:**
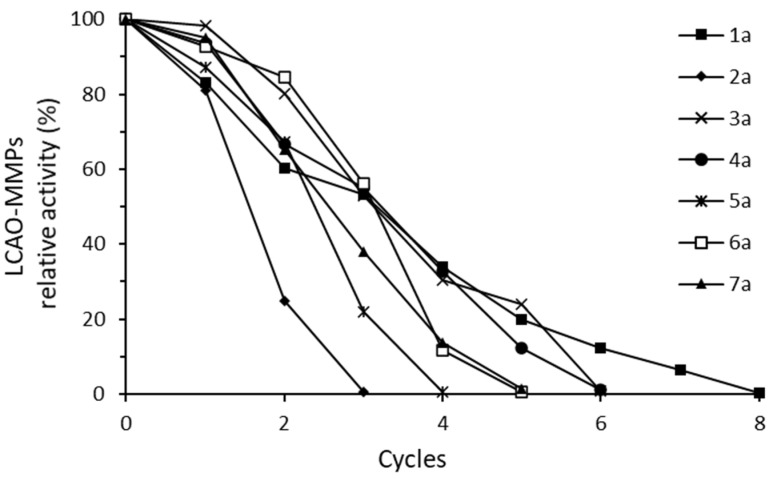
Reusability of LCAO immobilized on NH_2_-MMPs using compounds **1a**–**7a** as substrates in the presence of catalase. Data are presented as the mean of two experiments.

A strong indication of this behavior comes from the actual studies on the natural putrescine substrate (see Figure 4), where the increased stability of the enzyme allowed up to 15 reaction cycles. Based on both these observations and the kinetic parameters (Table 1), we decided to scale up the process using an initial 20 mM amine concentration (15 mL final volume) in the presence of catalase, then using the same condition at each reaction cycle. The results are shown in Figure 5 and Table 2.

LCAO-MMPs quantitatively convert all tested substrates, although the number of times they can be reused varies. The conversion at the end of each reaction cycle was evaluated by determining the concentration of aldehyde by means of a colorimetric assay with Purpald^®^. The overall conversion at the end of all cycles is about 90% for all tested substrates, with an aldehyde production ranging between 100 and 270 mg depending on the substrate used. These values show an increase in aldehyde yield up to 45 times higher than the previously published method with the free enzyme [28]. These yields can be further increased by excluding the last reaction cycle. In the last cycle, in fact, the residual relative activity is always very low while the quantity of amine is equal to that of the previous cycles (20 mM). This implies that the complete transformation is slower and that the aldehyde formed, over long reaction times, can generate by-products that reduce the overall yields. Therefore, excluding the last cycle, a purer product would be obtained, albeit in a smaller quantity. With this protocol, the aldehydes are obtained in aqueous media ready to be used in domino processes, without further extraction and purification.

**Scheme 2:**
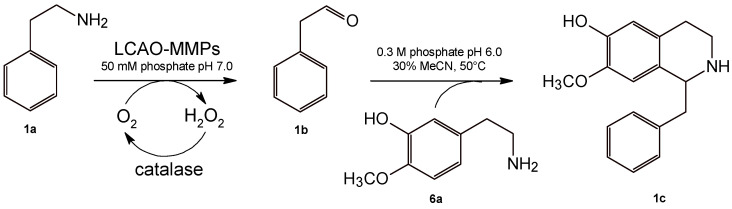
Biomimetic synthesis of compound **1c**.

In this regard, as a proof of concept, we used one of the aldehydes thus produced to synthesize a new non-natural benzylisoquinoline alkaloid. In nature, the first committed step for the synthesis of benzylisoquinoline alkaloids is the Pictet–Spengler cyclization between p-OH phenylacetaldehyde and dopamine, catalyzed by norcoclaurine synthase [36]. Pesnot et al. found that this reaction can be catalyzed by phosphate ions as well, although yielding the racemic product [37]. This can be a very convenient method for the synthesis of racemic benzylisoquinolines, as metabolic engineering and total chemical synthesis approaches, though occasionally applied, fail to meet industry standards in sustainability and efficiency. Other research groups have shown that the phosphate biomimetic catalysis may be suitable for the synthesis of different benzylisoquinolines, starting from dopamine (or analogs with a free OH in position 3) and a series of variously substituted aldehydes. Based on these findings, we decided to synthesize a benzylisoquinoline (**1c**), starting from **1b** (produced with LCAO-MMPs) and **6a** using phosphate ions as a catalyst (Scheme 2).

This reaction takes place in the same reaction pot as the aldehyde (after removing the immobilized enzyme), lowering the pH to 6 and increasing the ionic strength of the buffer (up to 0.3 M). Under these conditions, in the presence of acetonitrile as cosolvent and at a temperature of 50 °C, the Pictet–Spengler reaction takes place. In about 2 h the reaction is complete providing **1c** with a yield of 80%. Reaction yield was determined based on HPLC analyses, according to the method described in Section 4.

## 3. Conclusions

In this paper, we showed that it is possible to immobilize LCAO on different supports and that the best performance is obtained by using amine-functionalized magnetic particles. The enzyme immobilized on this support retains its activity, greatly improves its thermostability, and can be recycled numerous times with its natural substrate. Furthermore, immobilized LCAO can be used for biocatalytic applications. Specifically, this enzyme was used for the synthesis of a variety of aromatic aldehydes variously substituted starting from the corresponding amines. We showed that it is possible to scale up the process by recycling the immobilized enzyme a number of times and that it is possible to use the aldehydes thus produced for the synthesis of complex molecules, such as benzylisoquinoline alkaloids.

## 4. Materials and Methods

### 4.1. Chemicals

Reagents and solvents obtained from commercial suppliers were used without further purification. All chemicals were purchased from Merck KGaA (Darmstadt, Germany). CDCl_3_ (99.80% D) was obtained from Eurisotop (Saint Aubin, France).

### 4.2. LCAO Extraction and Purification

LCAO was extracted and purified by means of a chromatography-free protocol as reported by Di Fabio et al. [28]. Etiolated shoots (14 days old) were separated from the seed and roots and then shredded. Upon maceration in 0.3 M sodium chloride, the crude extract was filtered with the Sartolab^®^ Vacuum Filters System (Polyethersulfone, 0.22 μm) using highly pure diatomaceous earth (Sartoclear Dynamics^®^) as a filter aid (Sartorius). The extract was then subjected to tangential ultrafiltration using a Vivaflow 200 module (Sartorius), exchanging the buffer with 50 mM phosphate pH 6.5. The sample was heated for 15 min at 65 °C and centrifuged to remove the precipitated proteins. Enzyme purity was checked using SDS-PAGE.

### 4.3. LCAO Immobilization

LCAO immobilization was performed using different commercial solid supports (DEAE resin, amino- and carboxy-functionalized magnetic microparticles, chloromethyl latex, and amino-functionalized Turbobeads), using standard protocols.

The amount of immobilized enzyme was measured by the Bradford assay by determining the concentration of soluble protein before and after the immobilization procedure.

*DEAE fast flow resin:* 2.47 g of Sepharose fast flow resin (Ge Healthcare) were washed twice with deionized water, activated with 0.5 M sodium chloride, and equilibrated with 10 mL of 50 mM sodium phosphate pH 7.0. LCAO (180 U—3.2 mg) and 0.2% glutaraldehyde (final concentration) were then added, and the mix was left in gentle agitation for 1 h. The resin was extensively washed with 50 mM sodium phosphate pH 7.0, resuspended in the same buffer to a final volume of 6 mL, and stored at 4 °C.

*Chloromethyl latex beads:* 0.25 mL of chloromethyl latex beads solution (Thermo Fisher Scientific) were activated with 1 mL 25 mM MES buffer pH 6.0, then centrifuged at 6000 rpm for 20 min. The beads (30 mg) were resuspended in 1 mL 25 mM MES buffer pH 6.0 containing 1.7 mg of LCAO (94 U) and incubated overnight at room temperature under gentle stirring. Then, the particle solution was centrifuged for 15 min at 5000 rpm and the beads were washed twice with 2 mL PBS and stored at 4 °C in 2 mL of the same buffer containing 0.1% glycine and 1% Tween 20.

*Carboxy-functionalized magnetic microparticles (COOH-MMPs):* 0.2 mL of carboxy-functionalized magnetic microparticles (Merck KGaA) were washed with 2 mL 0.1 M MES buffer pH 5.3. Magnetic particles (4 mg) recovered using the LifeSep magnetic separation unit (Dexter Magnetic Technologies, Inc.) were resuspended in 2 mL of the same buffer containing EDC (10 mM) and left under gentle stirring for 30 min at room temperature. The activated particles were washed with 50 mM sodium phosphate pH 7.3. Immobilization took place by adding 2.7 mg of LCAO (150 U) dissolved in 2 mL of the washing buffer and leaving the particles under stirring at room temperature for 3 h. The mix was washed with phosphate buffer and then resuspended in 2 mL 50 mM sodium phosphate pH 7.0 containing 30 mM glycine (quencher) and 0.5% Tween 20. After 30 min under gentle stirring, the microparticles were recovered, washed with phosphate buffer, and stored at 4 °C in 50 mM sodium phosphate pH 7.0 containing 0.1% Tween 20.

*Turbobeads amine:* 30 mg of Turbobeads nanoparticles (Merck KGaA) were washed with deionized water, recovered using the LifeSep magnetic separation unit, resuspended in 1 mL 0.1 M MES buffer pH 6.0 and then sonicated for 1 min. The nanoparticles were extensively washed in the sonication buffer, resuspended in 2 mL of the same buffer containing 10% glutaraldehyde, and stirred for 1.5 h. The particles were then washed with 50 mM phosphate buffer pH 7.3 and incubated with 1.5 mg of LCAO (82 U) under gentle stirring at room temperature. After 3 h, the reaction was quenched exchanging the buffer with 50 mM phosphate pH 7.3 containing 30 mM glycine and 0.5% tween 20. After an additional 30 min, the particles were recovered, washed with phosphate buffer, resuspended in 2 mL 50 mM phosphate buffer pH 7.0 containing 0.1% tween 20, and stored at 4 °C.

*Amino-functionalized magnetic microparticles (NH_2_-MMPs):* 0.2 mL of amino-functionalized magnetic microparticles solution (Merck KGaA) were activated twice with 0.1 M MES buffer pH 6.0. After separation using the LifeSep magnetic separation unit, the particles (10 mg) were resuspended in the same buffer containing 10% glutaraldehyde and left under stirring for 1 h at room temperature. The magnetic beads were then recovered, washed with 50 mM phosphate buffer pH 7.3, and resuspended in 1 mL of the same buffer containing different amounts of LCAO (from 1 to 5 mg; 55–275 U). After 2.5 h, the reaction was quenched exchanging the buffer with 50 mM sodium phosphate pH 7.3 containing 30 mM glycine and 0.5% Tween 20. After further 30 min under gentle stirring, the magnetic particles were washed with 50 mM sodium phosphate pH 7 and 0.1% Tween 20 and stored at 4 °C in the same buffer.

### 4.4. LCAO Activity

Enzymatic activity of both free and immobilized LCAO was determined by a coupled diamine oxidase/peroxidase spectrophotometric assay. Relative activity was obtained by calculating the percentage of immobilized enzyme units versus the total units used in the immobilization reaction. 

The enzymatic assay was carried out in the presence of 10 mM putrescine at 25 °C and in 20 mM phosphate buffer pH 7.0. Enzymatic activity of LCAO immobilized on NH_2_-MMPs was also assayed using 2 U LCAO at different pH values (ranging between 5.5 and 8.0). The production of H_2_O_2_ was monitored following the increasing absorption at 515 nm, due to the coupling between 1 mM 4-aminoantipyrine (AAP) and 10 mM sodium-3,5-dichloro-2-hydroxybenzenesulfonate (DCHBS) catalyzed by horseradish peroxidase (2 U/mL). The initial rates of the reaction at different substrate concentrations (0.1–10 mM) were determined and the kinetic parameters were calculated by non-linear regression fitting the data to the Michaelis–Menten equation V = Vmax[S]/(Km + [S]). All curve fitting was carried out using Kaleidagraph software (Synergy Software, Reading, PA).

### 4.5. Enzymatic Synthesis of Aldehydes

The biocatalytic synthesis of the seven different aldehydes (compounds **1b**–**7b**) was carried out starting from the corresponding primary amines (compounds **1a**–**7a**) in the presence of LCAO immobilized on the surface of NH_2_-MMPs (LCAO-MMPs). A 20 mM solution of amine substrate was prepared in 50 mM sodium phosphate buffer pH 7.0 in the presence of 30 U of LCAO-MMPs and 250 U/mL of catalase to a final volume of 15 mL. At the end of each reaction cycle, the particles were recovered using the magnetic separation unit, extensively washed with phosphate buffer pH 7, and reused for the next reaction cycle.

The substrate consumption was monitored by GC/MS, whereas aldehyde formation was monitored by purpald^®^ assay and GC/MS.

GC/MS analysis: amine substrates (**1a**–**7a**) were analyzed as ethoxy carbonyl derivatives.

Derivatization with ethyl chloroformate (ECF) was conducted by adding to 50 μL of the reaction mixture, 25 μL of 7 M sodium hydroxide, and 25 μL ECF dissolved in 50 μL of dichloromethane. The biphasic system was stirred vigorously for 2 min, saturated with NaCl, and extracted with 125 μL of ethyl acetate. After centrifugation, 100 μL of the organic phase were analyzed by GC/MS using methyl-C17 as the internal standard. Aldehydes (**1b**–**7b**) were analyzed without derivatization: 100 μL of reaction mix were extracted with 400 μL of diethyl ether and directly analyzed by GC/MS. GC/MS analyses were performed with an Agilent 6850A gas chromatograph coupled to a 5973N quadrupole mass selective detector (Agilent Technologies, Palo Alto, CA, USA). Chromatographic separations were carried out with an Agilent HP-5ms fused silica capillary column (30 m × 0.25 mm id) coated with 5% phenyl–95% dimethylpolysiloxane (film thickness 0.25 μm) as a stationary phase. Injection mode: splitless at a temperature of 280 °C. Column temperature program: 70 °C for 4 min and then to 240 °C at a rate of 25 °C min^−1^ and held for 4 min. The carrier gas was helium at a constant flow of 1.0 mL min^−1^. The spectra were obtained in the electron impact mode at 70 eV ionization energy; ion source 280 °C; ion source vacuum 10^−5^ Torr. Mass spectrometric analysis was performed in the range m/z 50–500 at a rate of 0.42 scans s^−1^.

Purpald^®^ colorimetric assay: Aldehyde production was monitored by a colorimetric assay following the reaction between the newly synthesized aldehyde and 4-Amino-5-hydrazino-1,2,4-triazole-3-thiol (Purpald^®^) [28].

### 4.6. Biomimetic Synthesis of Compound ***1c***

Compound **1b** was synthesized starting from **1a** as described above. The immobilized enzyme was reused up to 8 times. After each reaction cycle, LCAO-MMPs were recovered and reused, while the newly synthesized aldehyde (15 mL 20 mM) was stored at −20 °C. At the end of the last cycle, the reaction fractions of each cycle were pooled, the pH was adjusted to 6.0, and the ionic strength of the buffer was increased to 0.3 M. Compound **6a** was added stoichiometrically to 1b and the phosphate mediated Pictet Spengler cyclization was carried out for 2 h at 50 °C in the presence of 30% acetonitrile.

The production of the benzylisoquinoline alkaloid (**1c**) was monitored by GC/MS after ECF derivatization (see above). Column temperature program: 70 °C for 1 min and then to 300 °C at a rate of 15 °C min^−1^ and held for 10 min.

### 4.7. Purification of Compound ***1c***

At the end of the reaction, the crude was allowed to cool at room temperature and extracted with dichloromethane. The combined organic layers were dried over Na_2_SO_4_, filtered, and concentrated under reduced pressure at 30 °C before being purified by preparative TLC using Macherey-Nagel TLC glass plates, silica gel layer, 1.0 mm, eluting with a mixture of n-hexane/EtOAc/MeOH (2/8/1 *v*/*v*) (Rf = 0.15) to give compound 1c as yellow solid.

### 4.8. NMR Analyses

Identification of product **1c** was performed by NMR analysis of the purified compound.

^1^H and ^13^C NMR (400.13 and 100.03 MHz) analyses were recorded with an Avance 400 spectrometer, equipped with a Nanobay console and Cryoprobe Prodigy probe (Bruker Italia S. r. l., Milano, Italy). About 20 mg of 1c were dissolved in 0.7 mL of CDCl_3_, transferred into an NMR tube, and analyzed. The resulting ^1^H NMR and ^13^C NMR spectra were processed using Bruker TOPSPIN TopSpin 3.5pl2 software.

### 4.9. High-Performance Liquid Chromatography Analyses

To calculate reaction yield, the crude was analyzed by HPLC. An HPLC-DAD apparatus (Perkin Elmer, Milan, Italy), equipped with an LC Series 200 pump, a Series 200 DAD, and a Series 200 autosampler, including Perkin Elmer TotalChrom software for data tracking was used. Analyses were performed at 280 nm with a Luna RP-18, 3µ column in isocratic elution consisting of acetonitrile (65%) and water acidified by 5% of formic acid (35%), at a flow rate of 1.0 mL min^−1^. The analyte **1c** was identified by comparing retention time to that of an authentic standard. Peak area was used to calculate analyte concentrations in the samples by reference to the standard curve attained by pure substance chromatography, under identical conditions. DAD response was linear within the calibration ranges with correlation coefficients exceeding 0.997.

## Figures and Tables

**Table 1:** Michaelis–Menten constant of free and immobilized LCAO towards **1a**–**7a** and putrescine.

Substrate	Km Free LCAO (mM) *	Km LCAO-MMPs (mM)
**putrescine**	0.27	0.36
**1a**	1.20	0.51
**2a**	0.60	0.79
**3a**	0.47	1.81
**4a**	5.37	4.74
**5a**	1.00	2.19
**6a**	1.58	0.64
**7a**	0.47	0.47

* [28].

**Table 2:** Reusability of LCAO immobilized on NH_2_-MMPs in the synthesis of **1b**–**7b**.

Product	Cycles	% Conversion	Total Amount (mg)
**1b**	8	92.85	267.40
**2b**	3	89.46	109.50
**3b**	6	91.50	247.05
**4b**	6	88.83	239.85
**5b**	4	89.93	194.25
**6b**	5	90.82	226.15
**7b**	5	89.48	222.80

## Data Availability

All data related to the manuscript are available in the manuscript and in the Supplementary Information in the form graphs, figures, and tables.

## Supplementary Materials

The following supporting information can be downloaded at: https://www.mdpi.com/article/10.3390/ijms23126529/s1. Ref [38] is cited in Supplementary Materials.
