# Supplementary material for: Immobilization of Lathyrus cicera Amine Oxidase on Magnetic Microparticles for Biocatalytic Applications
Source: Int J Mol Sci. 2022 Jun 10;23(12):6529. doi: 10.3390/ijms23126529 (PMC9223840; doi:10.3390/ijms23126529)
Supplement: Supplementary file 1 [file ijms-23-06529-s001.zip › ijms-1752776-supplementary.pdf]

SUPPLEMENTARY MATERIAL

Immobilization of *Lathyrus cicera* amine oxidase on magnetic microparticles for biocatalytic applications

Elisa Di Fabio[1], Antonia Iazzetti[2], Alessio Incocciati1, Valentina Caseli[3], Giancarlo Fabrizi[2] , Alberto Boffi[1,2], Alessandra Bonamore[1], and Alberto Macone [1,*]

[1] Affiliation 1; Department of Biochemical Sciences “Alessandro Rossi Fanelli”, Sapienza University of Rome, Piazzale Aldo Moro 5, 00185 Rome, Italy; elisa.difabio@uniroma1.it; alessio.incocciati@uniroma1.it; alberto.boffi@uniroma1.it; alessandra.bonamore@uniroma1.it; alberto.macone@uniroma1.it

[2] Affiliation 2; Center for Life Nano Science@Sapienza, Istituto Italiano Di Tecnologia, V.le Regina Elena 291, 00161 Rome, Italy; valentina.caseli@iit.it

[3] Affiliation 3; Department of Chemistry and Technology of Drugs, Sapienza University of Rome, Piazzale Aldo Moro 5, 00185, Rome, Italy; giancarlo.fabrizi@uniroma1.it; antonia.iazzetti@uniroma1.it

Table of Content

LCAO-MMPs ENZYMATIC ACTIVITY

Figure S1: Kinetic plots of immobilized LCAO

CARACTERIZATION OF COMPOUND 1c

Figure S2: Gas chromatogram and EI mass spectrum of **1c**

Figure S3: HPLC-DAD analysis of **1c**

Figure S4: ^{1}H, ^{13}C NMR spectra of **1c**

LCAO-MMPs ENZYMATIC ACTIVITY

Figure S1. Kinetic plots Kinetic plots of immobilized LCAO

All the kinetic parameters for compounds **1a**-**7a** were obtained from the Michaelis and Menten plots. Initial rates of reaction were determined at different substrate concentrations in the presence of LCAO-MMPs, at 25°C by means of a coupled LCAO-peroxidase spectrophotometric assay. The formation of the colored product was followed at 515 nm for 1 min. Each point is the mean of two experiments.

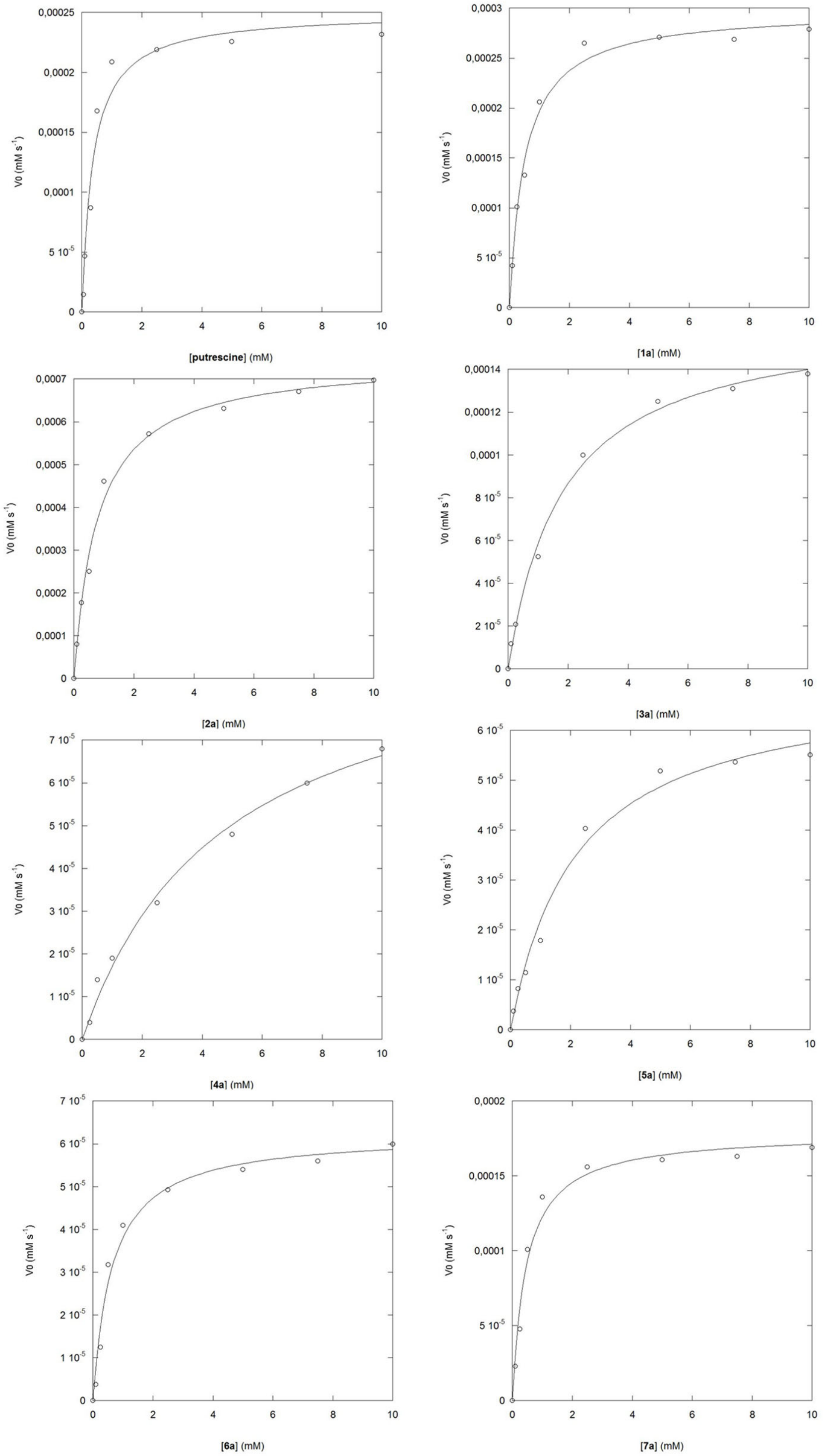

CARACTERIZATION OF COMPOUND 1c

Figure S2. Gas chromatogram and EI mass

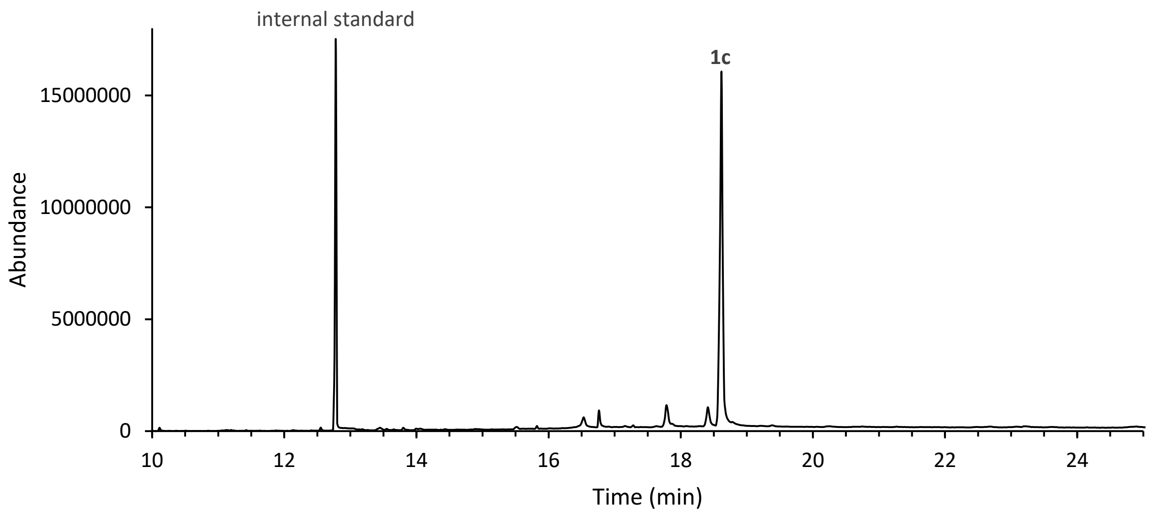

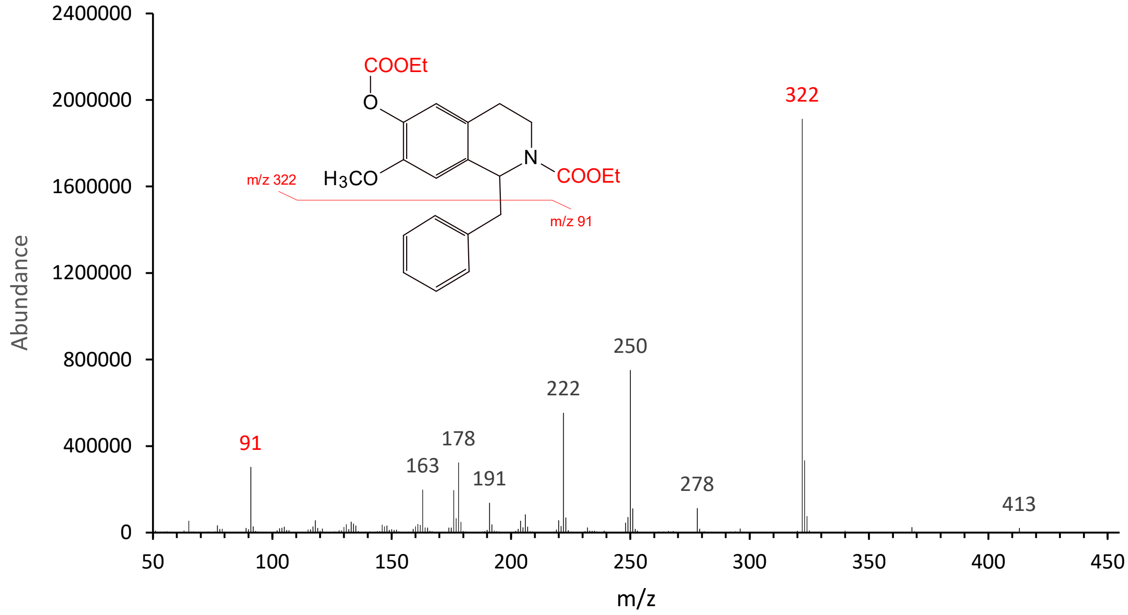

HPLC and NMR analyses of compound 1c

^{1}H NMR (400.13 MHz), and ^{13}C NMR (100.6 MHz) were recorded with a Bruker Avance 400 spectrometer, equipped with Nanobay console and Cryoprobe Prodigy probe. Splitting patterns are designed as s (singlet), dd (doublet of doublets), m (multiplet). IR spectra were recorded with a Jasco FT/IR-6800 spectrophotometer. Melting points were determined with a Büchi B-545 apparatus and are uncorrected.

1c: known compound; [1] yellow solid; mp: 151- 153 °C; IR (neat): 3305, 3055, 2916, 1604, 1532, 1494, 1334, 1209, 1125, 997 cm^{-1}; ^{1}H NMR (400.13 MHz) ($CDCl_3$): δ = 7.36 - 7.24(m, 5 H), 6.65 (s, 1 H), 6.59 (s, 1 H), 4.16 (dd, J_1 = 9.4 Hz, J_2 = 4.4 Hz, 2 H), 3.8 (s, 3 H), 3.24- 3.17 (m, 2 H), 2.97 – 2.88 (m, 3 H), 2.77 – 2.65 (m, 2 H); ^{13}C NMR (100.6 MHz) ($CDCl_3$): δ = 144.8 (C), 144.1 (C), 139.1 (C), 129.9 (C). 129.4 (CH), 128.6 (CH), 128.0 (C), 126.5 (CH), 114.8 (CH), 108.8 (CH), 56.9 (CH), 55.9 (CH_3), 42.9 (CH_2), 40.6 (CH_2), 29.2 (CH_2).

HPLC- DAD Analysis

The calibration curve was built and used for the quantitation of **1c** in the reaction mixture, using a sample of the same purified compound (R^2 = 0.997), as the reference standard.

Figure S3. **HPLC-DAD analysis of compound 1c.** The measurement was performed at 280 nm: chromatogram overlapping of authentic standard (upper curve) and analyte (lower curve).

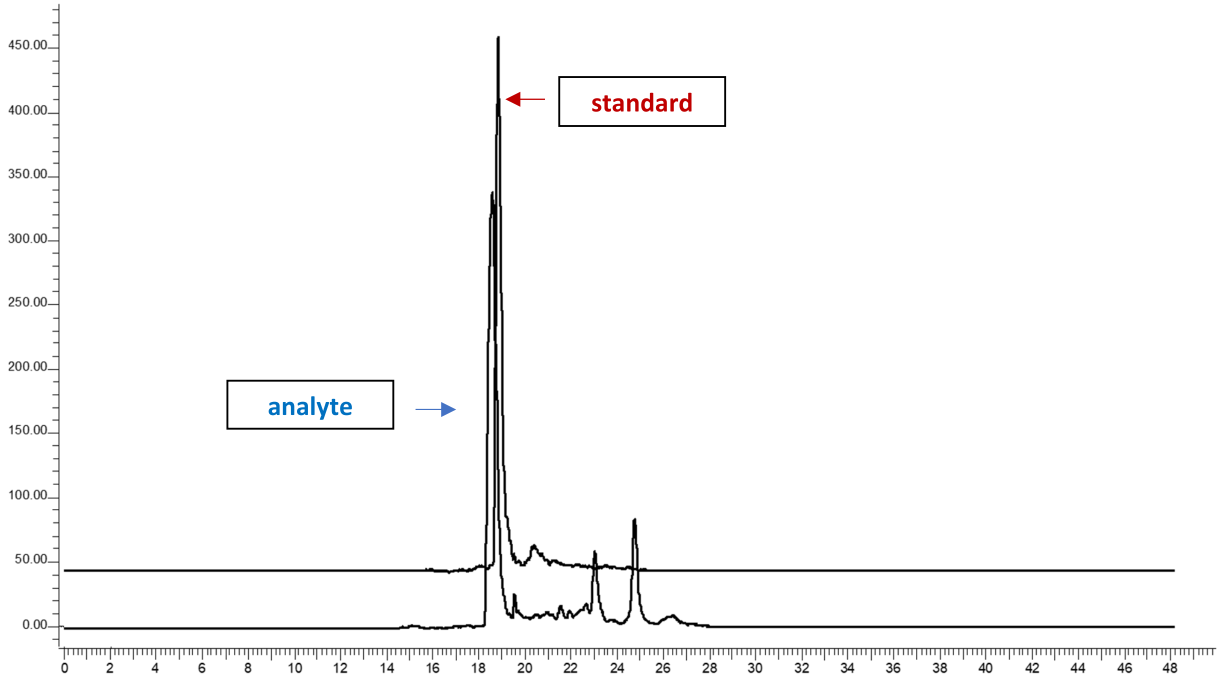

Figure S4: ^{1}H, ^{13}C NMR spectra of **1c**

^{1}H NMR (400.13 MHz), $CDCl_3$

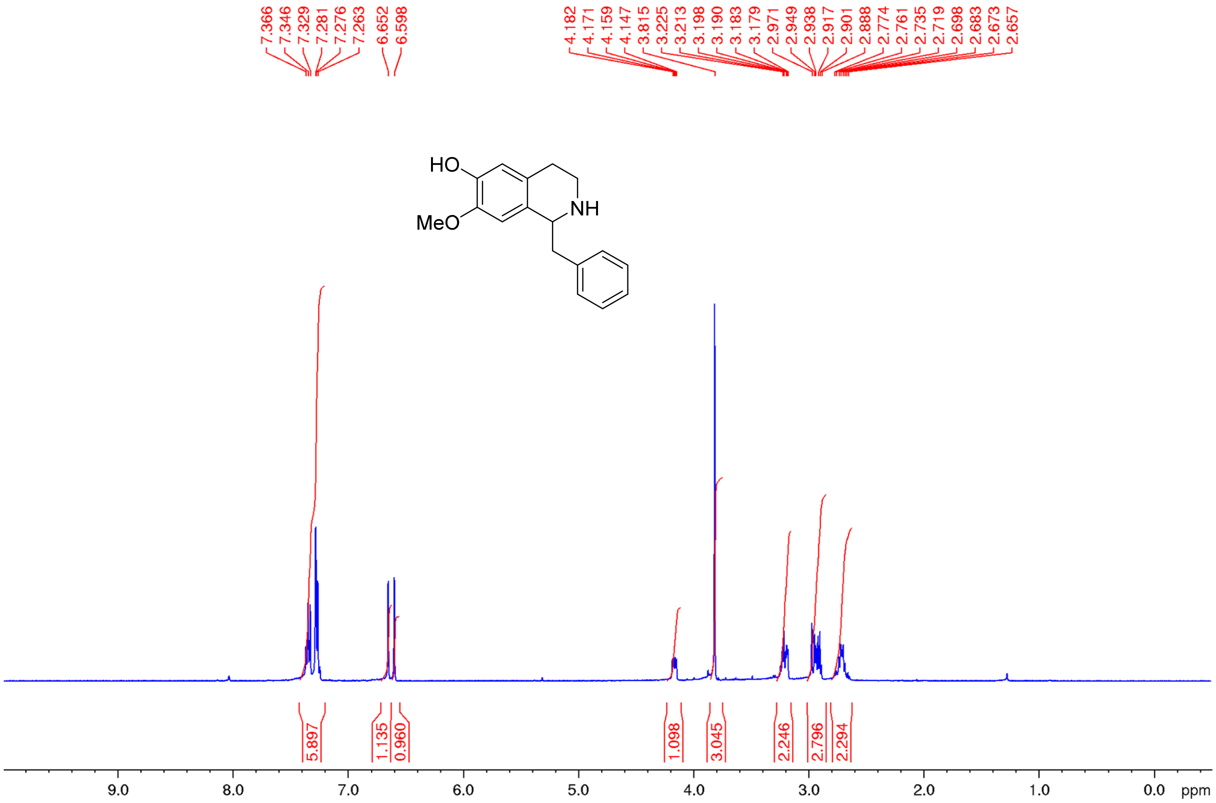

^{13}C NMR (100.6 MHz), $CDCl_3$ (1c)

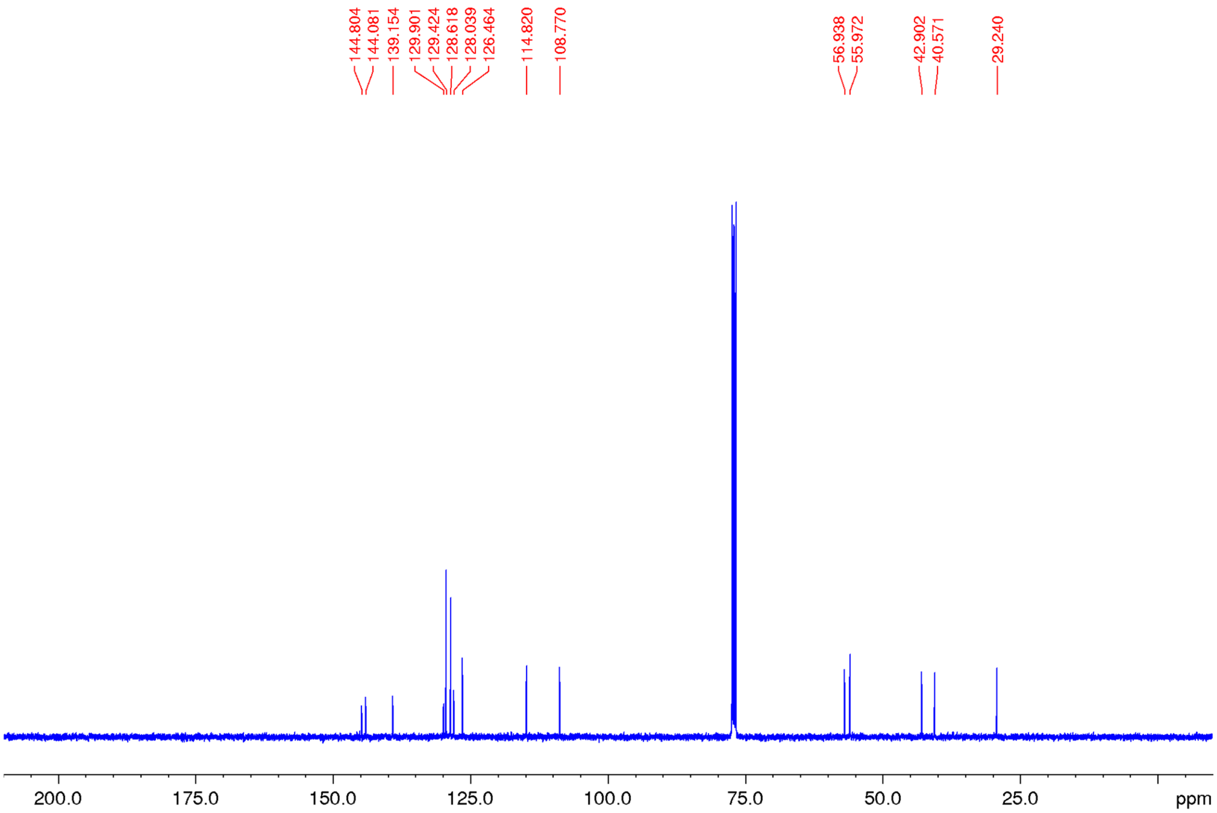

^{13}C NMR - DEPT, $CDCl_3$ (1c)

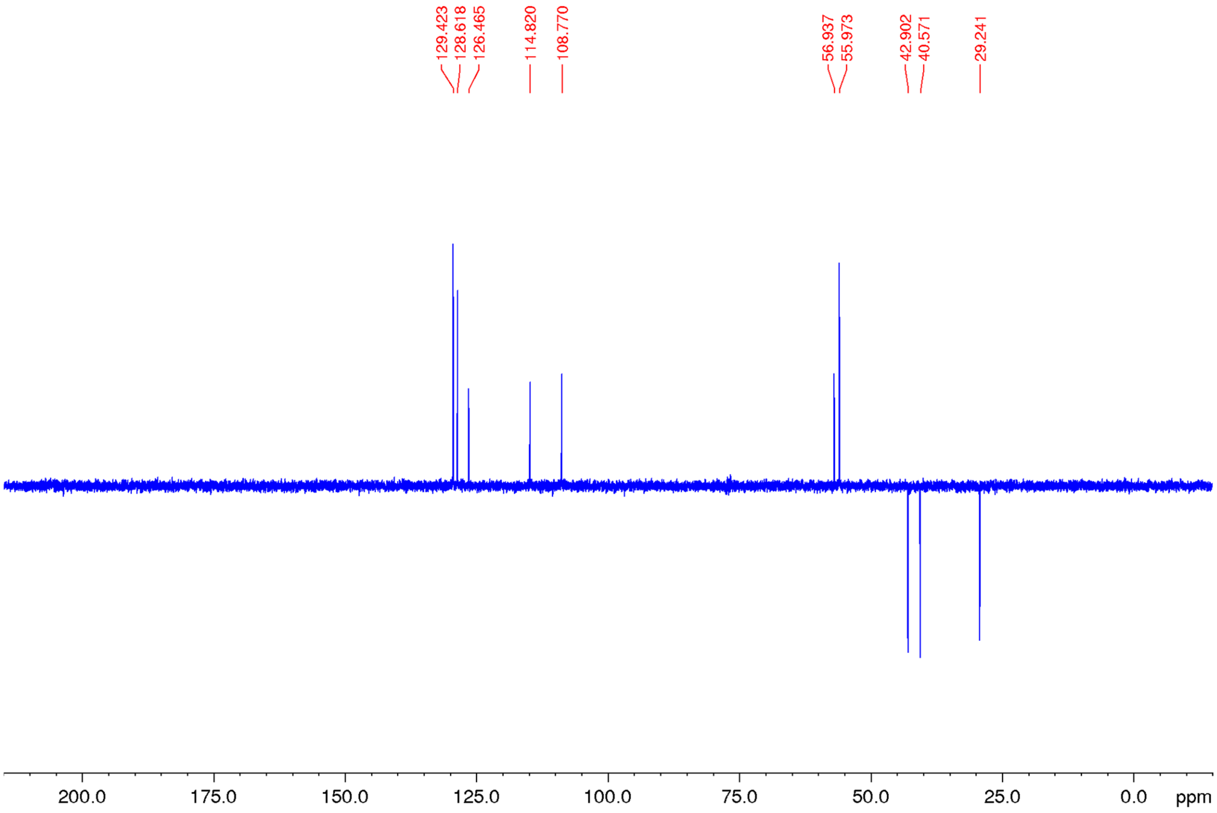

References

[1] Andreu, I.; Cortes, D.; Protais, P.; Cassels, B. K.; Chagraoui, A.; Cabedo, N., Preparation of dopaminergic N-alkyl-benzyltetrahydro-isoquinolines using a 'one-pot' procedure in acid medium. *Bioorganic & Medicinal Chemistry* **2000,** *8*, 889-895.
